# Coincidence of pheromone and plant odor leads to sensory plasticity in the heliothine olfactory system

**DOI:** 10.1371/journal.pone.0175513

**Published:** 2017-05-03

**Authors:** Elena Ian, Nicholas H. Kirkerud, C. Giovanni Galizia, Bente G. Berg

**Affiliations:** 1 Norwegian University of Science and Technology (NTNU), Department of Psychology, Trondheim, Norway; 2 Universität Konstanz Fachbereich Biologie, Konstanz, Germany; INRA-UPMC, FRANCE

## Abstract

Male moths possess a highly specialized olfactory system comprised of two segregated sub-arrangements dedicated to processing information about plant odors and pheromones, respectively. Communication between these two sub-systems has been described at the peripheral level, but relatively little is known about putative interactions at subsequent synaptic relays. The male moth faces the challenge of seeking out the conspecific female in a highly dynamic odor world. The female-produced pheromone blend, which is a limited resource serving as guidance for the male, will reach his antennae in intermittent pockets of odor filaments mixed with volatiles from various plants. In the present study we performed calcium imaging for measuring odor-evoked responses in the uni-glomerular antennal-lobe projection neurons (analog to mitral cells in the vertebrate olfactory bulb) of *Helicoverpa armigera*. In order to investigate putative interactions between the two sub-systems tuned to plant volatiles and pheromones, respectively, we performed repeated stimulations with a selection of biologically relevant odors. We found that paired stimulation with a plant odor and the pheromone led to suppressed responses in both sub-systems as compared to those evoked during initial stimulation including application of each odor stimulus alone. The fact that the suppression persisted also after pairing, indicates the existence of a Hebbian-like plasticity in the primary olfactory center established by temporal pairing of the two odor stimulation categories.

## Introduction

The evidence of phylogenetically highly conserved principles of the olfactory system allows exploration of its basic mechanisms in many different animal models including insect species. Studies of neural principles have been performed at different levels of the insect olfactory pathway, from sensory neurons to the higher centers of the brain. Here, we study the primary olfactory center in the insect brain, the antennal lobe (AL), which is the analogue to the vertebrate olfactory bulb [1]. The AL is the target area of the numerous olfactory sensory neurons (OSNs) located on the antenna. Like in vertebrates, many insects possess two main categories of OSNs, one being devoted to recognize general odorants and the other specialized for detection of sexual pheromones. Noctuid moths, for example, are reported to hold one group of OSNs tuned to plant odors and one male-specific category specifically tuned to female-produced substances. These two types of OSNs project to different regions in the AL: the plant odor neurons end up in the numerous "ordinary glomeruli" (OG), whereas the male-specific neurons terminate in a few enlarged glomeruli forming the macroglomerular complex (MGC; reviewed by Hansson and Anton [2]). Also the subsequent areas of the male olfactory pathway, the calyces and the lateral horn (LH), include segregated output regions for projection neurons (PNs) originating from the OG and the MGC, respectively [3–5].

The presence of distinct anatomical subsystems, one for female-produced cues and one for plant odors, indicates the expediency of separating the signal processing linked to each stimulus category. On the other hand, several studies have reported about interactions between the two subsystems–which seem highly relevant based on the fact that the pheromone generally will appear together with or on the background of particular plant odors. The findings from these kinds of investigations are partly inconsistent, however. Three previous studies on heliothine moths have explored how plant odors affect pheromone responses at the level of OSNs, one reporting about synergism [6] and the other two about suppression [7, 8]. Suppression of the pheromone response when adding a particular plant odor to the key compound was also found in a more recent study on the moth, *Agrotis ipsilon*, not only in the OSNs but also in the PNs [9]. An investigation on the silk moth, *Bombyx mori*, however, demonstrated that PNs tuned to the main pheromone component, bombykol, enhanced their response when a particular plant odor was presented together with the pheromone compound [10]. The same plant-pheromone mixture did not affect the response of plant odor PNs in an equivalent manner, however. Finally, in a study on the codling moth, *Cydia pomonella*, not only MGC-PNs but also neurons confined to OG were reported to display both synergistic and inhibitory responses during stimulation with various combinations of plant-pheromone mixtures [11]. Data from behavioral experiments in other moths species have demonstrated that the presence of host plant volatiles promote detection of female pheromones and significantly influence the male sexual response [12–14]. Among the relatively few publications testing the effect of pheromones on the plant odor system is a study on *Agrotis ipsilon* reporting about additive responses to the mixtures of the pheromone and a plant odorant [15].

Olfactory information from the AL is conveyed to the second order areas, the calyces of the mushroom body and the LH, via three main antennal lobe tracts (ALTs): the medial, mediolateral, and lateral ALTs. In a recent study of the heliothine moth, we demonstrated that the calyces get input mainly from the uni-glomerular PNs belonging to the medial ALT [16]. This kind of arrangement allowed us to measure odor-evoked activity from the medial-tract PNs specifically by applying the calcium-sensitive dye fura2 into the calyx. In the study presented here, we have therefore explored interactions between the two systems dedicated to plant odors and the pheromones, respectively, by performing calcium imaging measurements from the uni-glomerular PNs in the heliothine species, *Helicoverpa armigera*.

Another advantage of studying *H*. *armigera* is that several biologically relevant input cues from the periphery to the AL are characterized in this species. Each of the two principal pheromone compounds, the primary constituent, *cis*-11-hexadecenal (Z11-16:AL), and the secondary, *cis*-9-hexadecenal (Z9-16:AL), is detected by one group of male-specific OSNs [17]. Natural plant odor mixtures, e.g. sunflower (*Heliopsis helianthoides*) headspace or ylang-ylang (*Cananga odorata*) on the other hand, comprise numerous odorant ligands for OSNs, several of which have been identified by gas chromatography [18]. All the previous studies on pheromone-plant interaction mentioned above included single odorants as test stimuli. In the present work we used two natural plant odor mixtures highlighting the idea that these kinds of odor blends may elicit neural activity corresponding more closely to that occurring under natural conditions. The use of natural mixtures, in contrast to monomolecular odorants, may provide more relevant results reflecting a full “picture” of neural coding mechanisms that underlie odor-driven behavior [19]. A recent publication indicates that doses exceeding naturally occurring amounts of plant odors might lead to wrong conclusions about their behavioral effects [20]. We therefore used substantially lower concentrations of the plant odor stimuli than many of the previous studies.

Calcium imaging measurement of the neuronal activity demonstrated an overall suppression effect during combined stimulation with plant odor and pheromone, in the OG as well as the MGC. Moreover, repeated stimuli led to increasing and persistent suppression, both in the OG and the MGC, indicating neural plasticity in the AL network.

## Methods

### Insects

We used 3–5 days old virgin male *H*. *armigera* (Lepidoptera: Noctuidae; Heliothinae) for the experimental work. Pupae were delivered by Henana Jiyuan Baiyun Industry Ca., Ltd. The pupae/insects were kept on a phase-shifted LD 14–10 h at 24°C and 60% relative humidity with *ad libitum* access to 10% sucrose solution. Adults, 3–7 days old, were used for the experiments. According to Norwegian law of animal welfare there are no restrictions regarding experimental use of Lepidoptera.

### Staining

**Fig 1:**
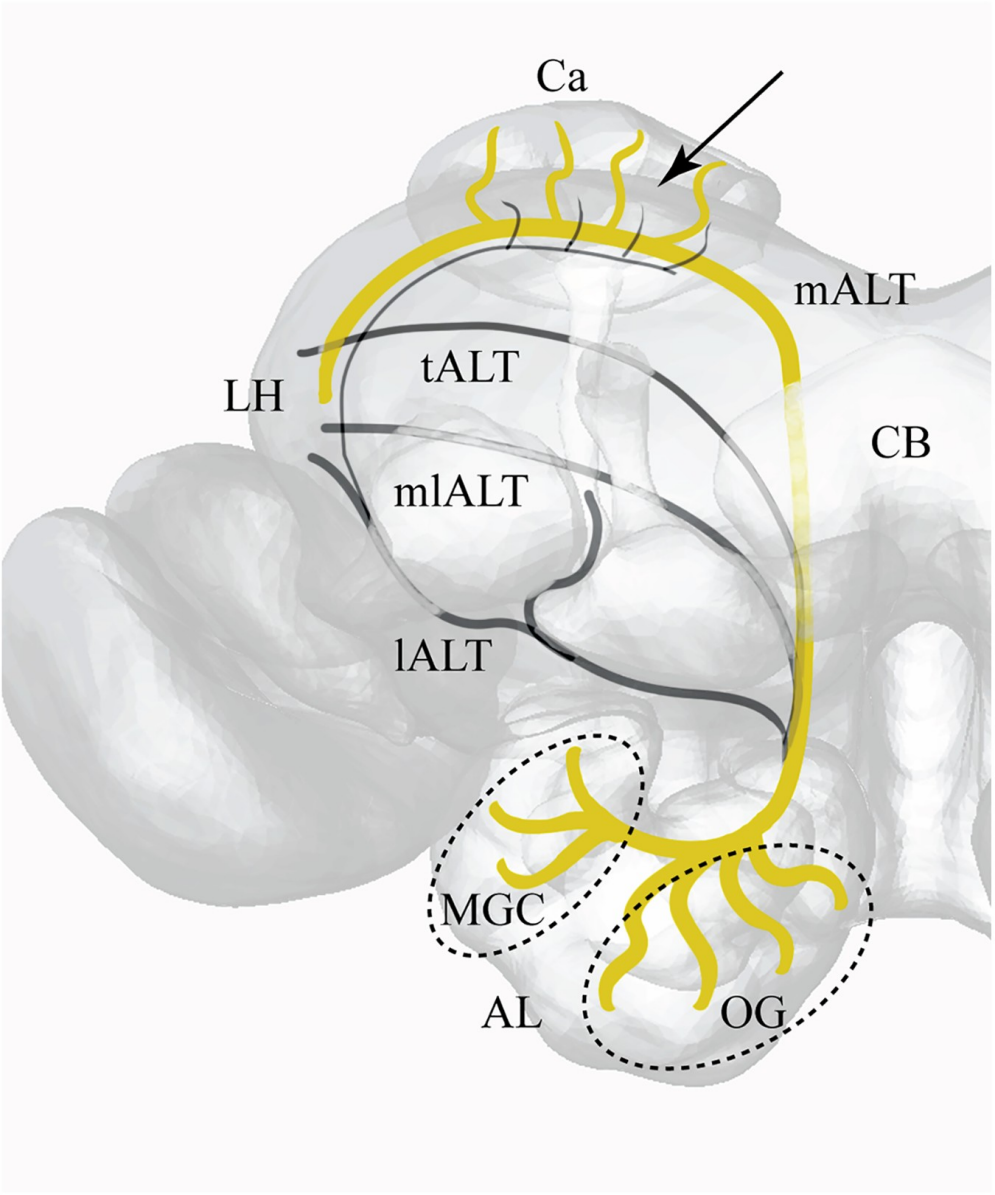
Projection view of a 3D-model of the right hemisphere with antennal lobe tracts and major brain areas indicated. Arrow indicates insertion point for fluorescent dye into the calyx. AL (Antennal Lobe), Ca (Calyx), CB (Central Body), LH (Lateral Horn), mALT (medial AL tract), tALT (transverse AL tract), mlALT (mediolateral AL tract), lALT (lateral AL tract), MGC (Macro Glomerular Complex), OG (Ordinary Glomeruli).

The moth was placed inside a small plastic tube with the exposed head restrained by wax (Kerr Corporation, Romulus, MI). The head capsule was opened; membranes and trachea covering the mushroom body calyx were gently removed. Selective staining of PNs was achieved as described for other species [21]. A glass electrode (pulled with a P-97 micropipette puller SUTTER Instruments) loaded with fura-2 dextran dye (10,000 MW, in 2% BSA; Molecular Probes) was inserted into the medial–lateral part of the calyx to stain projection neurons of the mALT (Fig 1).

Then the insect was kept in the dark either at room temperature for 4 hours or at 4°C in a refrigerator overnight. Just prior to imaging tissue covering the ALs was gently removed, and muscles enabling movement of the antennae were detached before the antennae were lifted up by needles. Saline solution (in mM: 150 NaCl, 3 CaCl_2_, 3 KCl, 25 Sucrose, and 10 N-tris (hydroxymethyl)-methyl-2-amino-ethanesulfonic acid, pH 6.9) was constantly applied to the brain.

### Imaging

In vivo calcium imaging recordings were done with an epifluorescent microscope (Olympus BX51WI) equipped with a 20x (NA 1.00) water immersion objective (Olympus XLUMPlanFLN). Images were acquired by a 512x512 pixel 16-bit electron-multiplying CCD camera (Photometrics Evolve 512). The preparation was alternately excited with 340 and 380 nm monochromatic light (TILL Photonics Polychrome V), and data were acquired ratiometrically. A dichroic mirror (420nm) together with an emission filter (490-530nm) was used to separate the excitation and emission light. We used 2x2 binning on chip (pixel size ~1x1μm), and each recording consisted of 150 double frames at a sampling frequency of 10 Hz with 25–35 ms and 10–15 ms exposure times for light of the two respective wavelengths. Each recording was of 15 s duration, started 3 s prior to onset of the odor stimulus, and the time interval between each recording was 60 s.

### Odor stimulation

**Fig 2:**
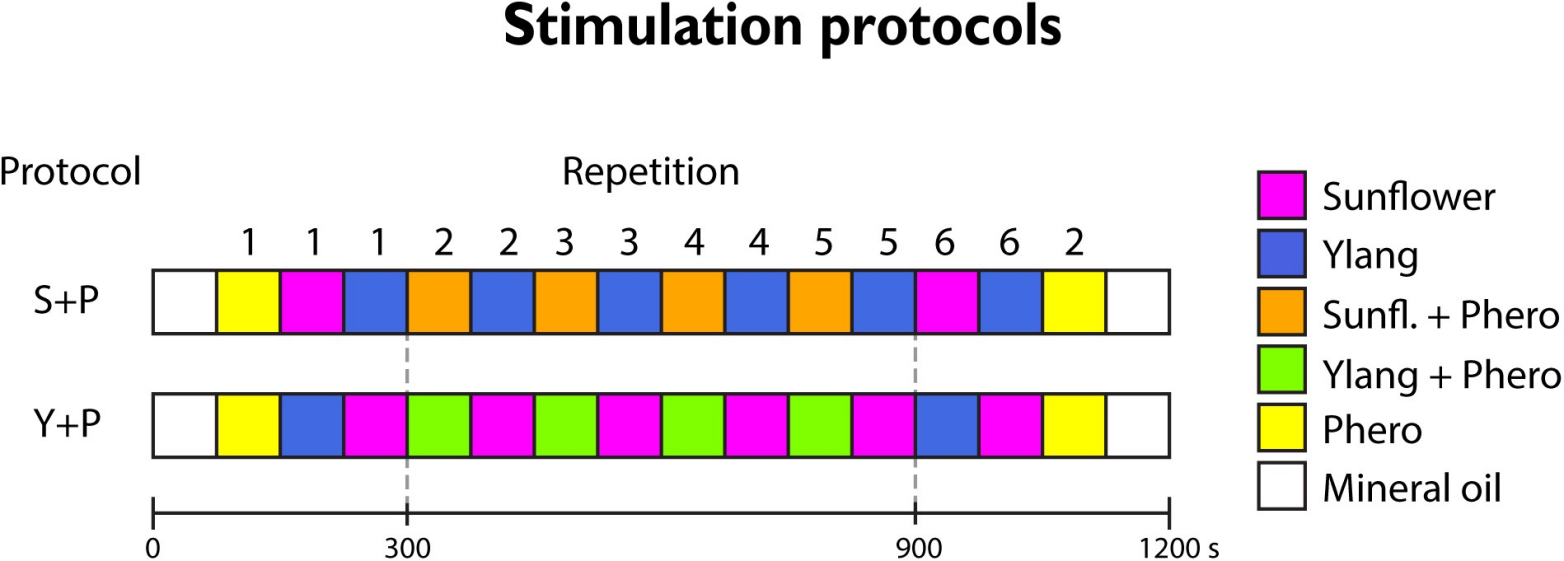
Sequence of odor stimuli for the 2 protocols. Colored squares indicate recordings of individual stimuli. Each stimulus lasted 2 s, with the recording starting 3 s before odor onset and lasting for 15 s. Time between recordings (and each odor stimulus onset) was 75 s. The time scale is nonlinear due to the breaks between recordings, and indicate elapsed time after the initial presentation of the individual odors (300 s) and after the repeated alternate sequence of the phero-paired and unpaired plant odor presentations (900 s).

The primary and secondary sex pheromone of *H*. *armigera*, Z11-16:AL and Z9-16:AL, respectively, were supplied by Pherobank, Wijk bij Duurstede, The Netherlands. The two pheromone constituents were solved in mineral oil (Acros Organics) in a 22:1 proportion (cumulative final concentration 0.1%) to resemble the natural blend released by females in the wild, henceforth referred to as “the pheromone” for simplification. Headspace of sunflower solved in hexane and further diluted in mineral oil (1%) and ylang-ylang essential oil further dissolved in mineral oil (1%) were used as complex plant blends. Ten μl of each of the three odor solutions was applied to a filter paper inside a 150 mm glass Pasteur-pipette. Odor pulse stimulation of 2 s duration was carried out by a stimulus controller (SYNTECH CS-55), by which humidified charcoal filtered air was delivered through PTFE tubes. The insect was subjected to one of two stimuli sequences including either sunflower (SF) or ylang-ylang (YL) paired with the pheromone (F) (Fig 2).

In each stimulation sequence, the first and the last stimulus included the plant blend without pheromone. The onset of one recording to onset of the next was set to 75 s. Prior to the first stimulus repetition each of the plant blends, plus the pheromone, were presented to evaluate the quality of the preparation. However, these responses were not included in the analysis due to the high variability that is normally observed in initial odor responses [22].

### Analysis

Recordings were acquired with Live Acquisition V2.3.0.18 (TILL Photonics) and imported in KNIME Analytics Platform 2.12.2 (KNIME GmbH, Konstanz, Germany) where ImageBee neuro plugin was used to construct antennal maps and glomerular time series. Statistical analyses were conducted in R (R Core Team 2015). Relative change in fluorescent levels for each glomerulus was calculated as F_340_/F_380_, followed by a baseline shift to 0 by subtracting the average baseline activity obtained in frames 10–30 (2 s period prior to stimulus onset). Response strength was calculated by averaging this relative change in signal over frames 40–70 (3 s period starting 100 ms after stimulus onset). Since the response strength variable meets the requirements of a normal distribution (S1 Fig) we used parametric t-tests to compare pairwise observations of the first and last stimulus repetition for each odor. Glomeruli belonging to the MGC were readily identified across individuals. The OG which comprised the rest of the AL, however, varied in size and position to such an extent across individuals that identification at the unit level was unfeasible with the methods at hand. Thus, we operated with an average response strength pooled over all responding OG. An OG was defined as responding if its relative signal change during the response window (frame 40–70) was larger or smaller than the mean ± 3xSD of its baseline activity (frame 10–30). Change in response strength over repetitions in the different sequence series was assessed by fitting linear mixed models (lme function in R nlme package) with *Repetition* as fixed effect, and *Subject* as random effect to account for repeated measurements.

To further investigate response patterns of the PNs comprising the OG, we performed a principle component analysis (PCA) (prcomp function in R base package). In the PCA, principal components were calculated on the hyper-volume spanned by all the analyzed OG for each of the two groups (sunflower + pheromone and ylang-ylang + pheromone). Response strength from the 1^st^, 4^th^, and 6^th^ repetition of each odor was used, corresponding to before, during, and after pheromone-pairing. Loadings of each odor response onto the two first principle components were compared to quantify divergence or convergence in the PN response patterns.

## Results

Based on labeling experiments including application of the calcium-sensitive dye into the calyx, we were able to measure neural activity mainly in one distinct population of AL output neurons in the moth brain, i.e. the uni-glomerular PNs confined to the mALT. The principal goal of our investigation was to explore interactions between pheromone and plant odor responses in the AL during antennal stimulation with a combination of a plant odor blend, sunflower or ylang-ylang, and the binary pheromone mixture. In order to study how plant odors affect pheromone responses and vice versa we used two stimulation protocols; in each, one plant odor blend was mixed with the pheromone and repeatedly presented to the moth in an alternate sequence including the other plant odor blend presented alone (Fig 2).

**Fig 3:**
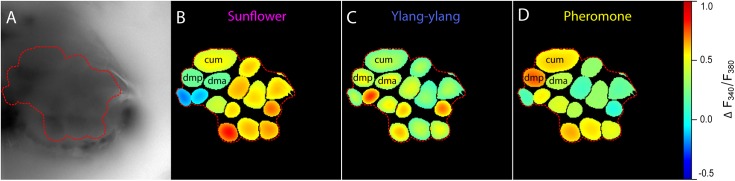
Automatic AL map reconstruction and response patterns upon odor stimulation. A: Unprocessed image of a representative AL stained with Fura-2, and with red border circumventing the area of recognized glomeruli. B-D: Heat maps of responses to sunflower, ylang-ylang and pheromone respectively (@ frame 50, 2 s after odor onset) in recognized regions of interest based on correlated activity in neighboring pixels. In each individually recorded antennal lobe the three glomeruli that made up the MGC were identified: cumulus (cum), dorsomedial anterior (dma) and dorsomedial posterior (dmp). The ordinary glomeruli comprised the rest of the AL.

Initial stimulation with each of the two plant odor blends alone (sunflower or ylang-ylang) typically induced glomerular activation patterns with considerable overlap and similar response dynamics in 3–6 OG, while calcium responses in 1–2 glomeruli significantly differed (example in Fig 3A–3C). Stimulation with the pheromone alone resulted in excitatory responses mainly in the three MGC glomeruli (Fig 3D).

### Pheromone and plant-odor interaction causes suppression in the AL projection neurons

**Fig 4:**
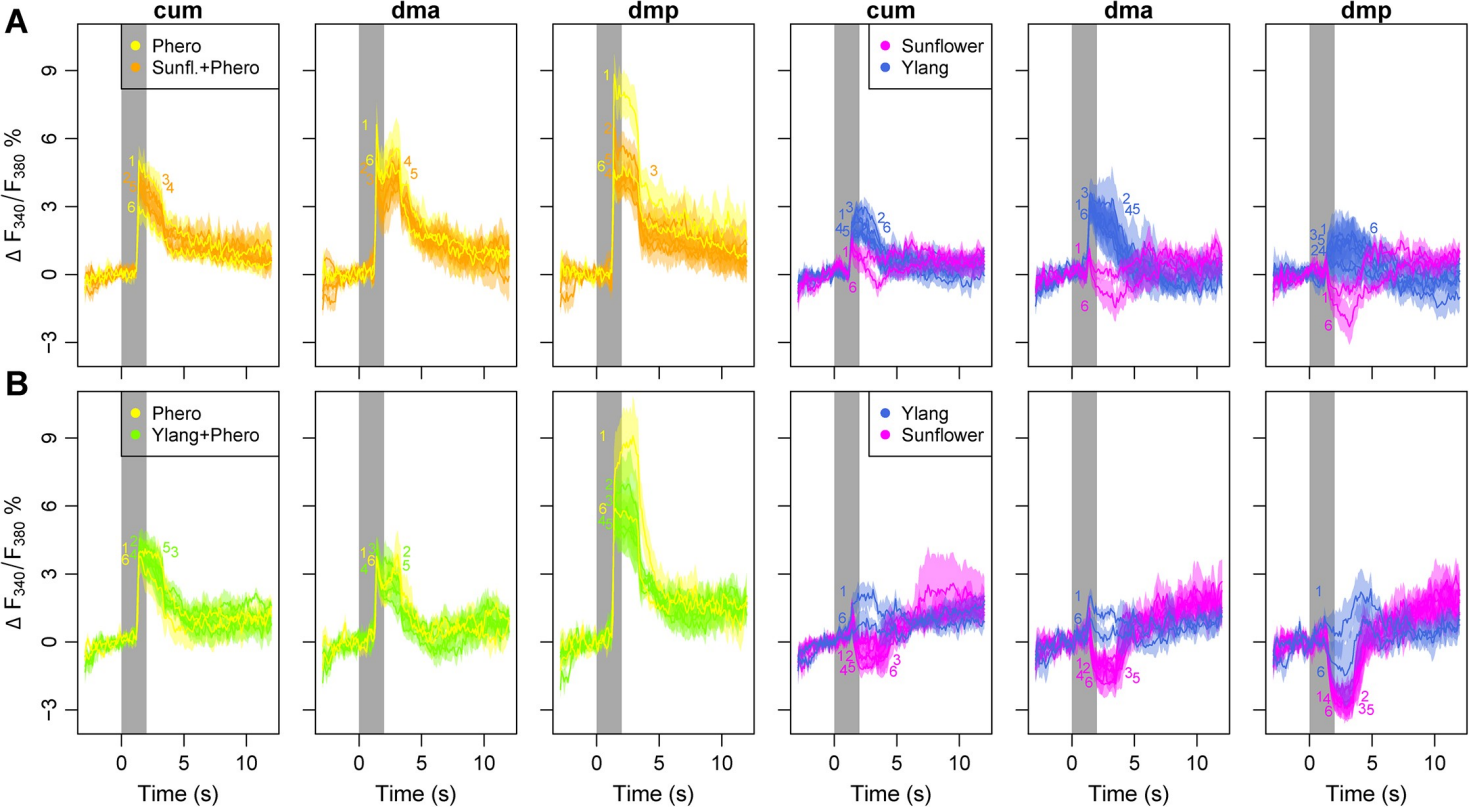
Calcium traces of the MGC glomeruli (cum, dmp, dma) during stimulation with a combination of sunflower/ylang-ylang and pheromones. Both plant blends were presented 6 times in an alternated sequence (see methods) to each animal. One group experienced pairing of sunflower with pheromones and ylang-ylang as unpaired (top row, A), while in the other group ylang-ylang was pheromone-paired and sunflower was unpaired (bottom row, B). All traces show mean ± SE for n = 8 and n = 5 ALs in the 2 groups, respectively. The grey column indicates stimulus window, and colored integers indicate repetition aligned with either the max or min of each trace, depending on whether the signal was excitatory or inhibitory.

**Fig 5:**
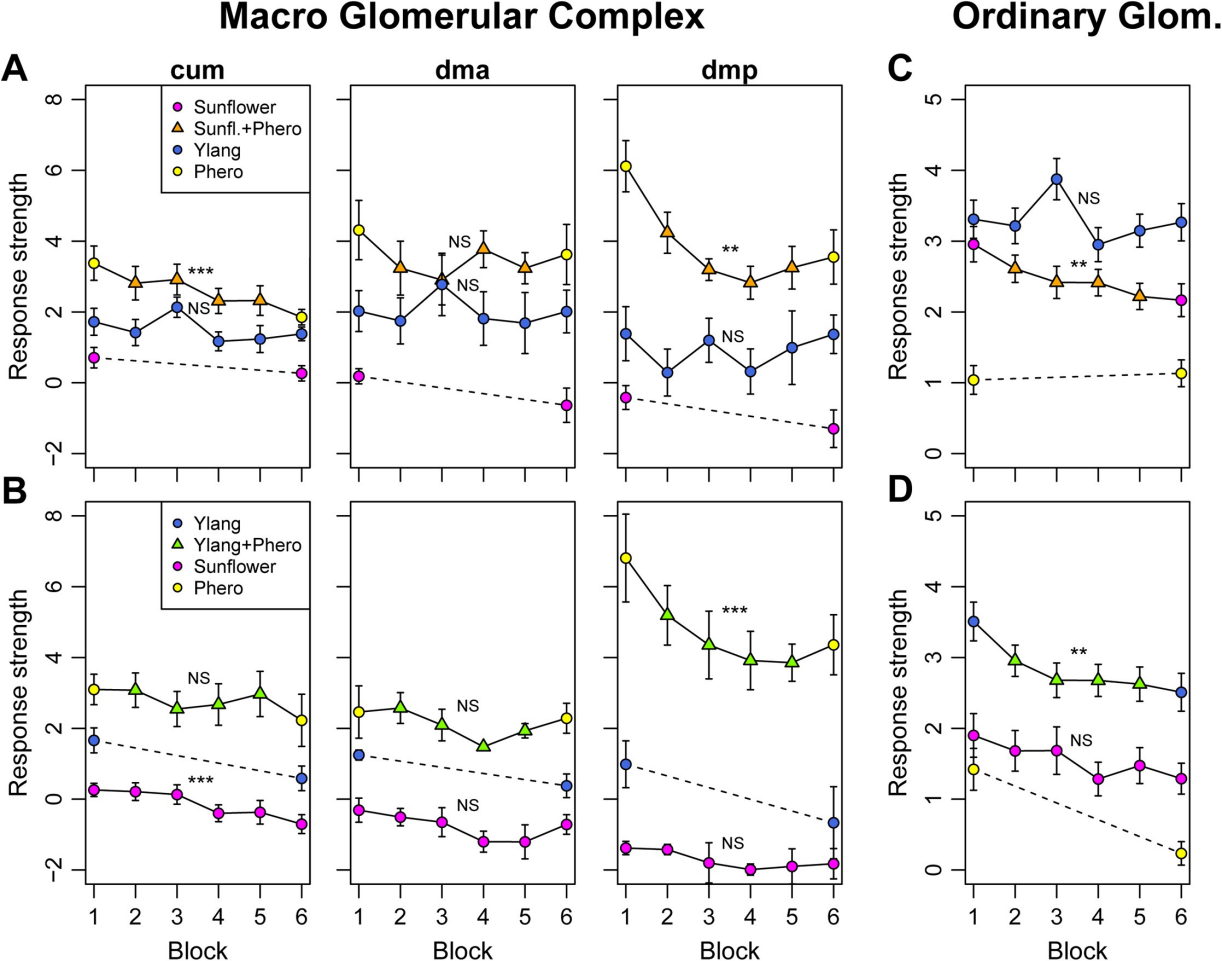
**Response strengths to repeated alternate stimulation with the two plant blends and pheromones reveal suppression effects in both MGC (A and B) and OG (C and D)**. Suppression was present for either of the two plant blends when accompanied with pheromones (sunflower in top row and ylang-ylang in bottom row). Presence of plant odor generally reduced the response to pheromones in MGC (A and B), while presence of pheromone reduced the responses to the plant odor in the OG (C and D). Response strengths represent the ΔF_340_/F_380_ averaged over the response period (frame 40–70) for the respective glomeruli. Mean ± SE presented from n = 8 and n = 5 AL’s for the two groups, respectively. Asterisks indicate significant changes in response strength over repetitions for each of the stimulus series (LME; ‘*’ p < 0.05, ‘**’ p < 0.01, ‘***’ p < 0.001, ‘NS’ Not Significant).

Due to the limited number of glomeruli comprising the MGC, and its location dorso-medially in the AL, close to the entrance of the antennal nerve, it was possible to identify each individual unit. The relatively low variance in calcium responses of the corresponding unit across individuals allowed a split-view analysis of the cumulus, the dorsomedial anterior, and the dorsomedial posterior glomerulus, respectively (Figs 4 and 5).

For the MGC, we observed that sunflower elicited a sharp but weak and transient excitation followed by a prolonged inhibition in all three glomeruli, while ylang-ylang elicited a weak excitatory response (peak at 20–40% of the pheromone response peak) without the inhibition (Fig 4).

The pheromone-plant mixture elicited a strong response, albeit reduced compared to the pheromone alone in the cumulus and dmp for sunflower (Figs 4A and 5A), and in the dmp for ylang-ylang (Figs 4B and 5B). This reduction was consistent for all four repetitions of the pheromone-plant odor mix (repetition 2–5), and indicated that the plant odors had a suppressive effect on the MGC. However, the response strength was not reduced in the cumulus and the dma for ylang-ylang, nor in the dma for sunflower (Fig 5A and 5B).

**Fig 6:**
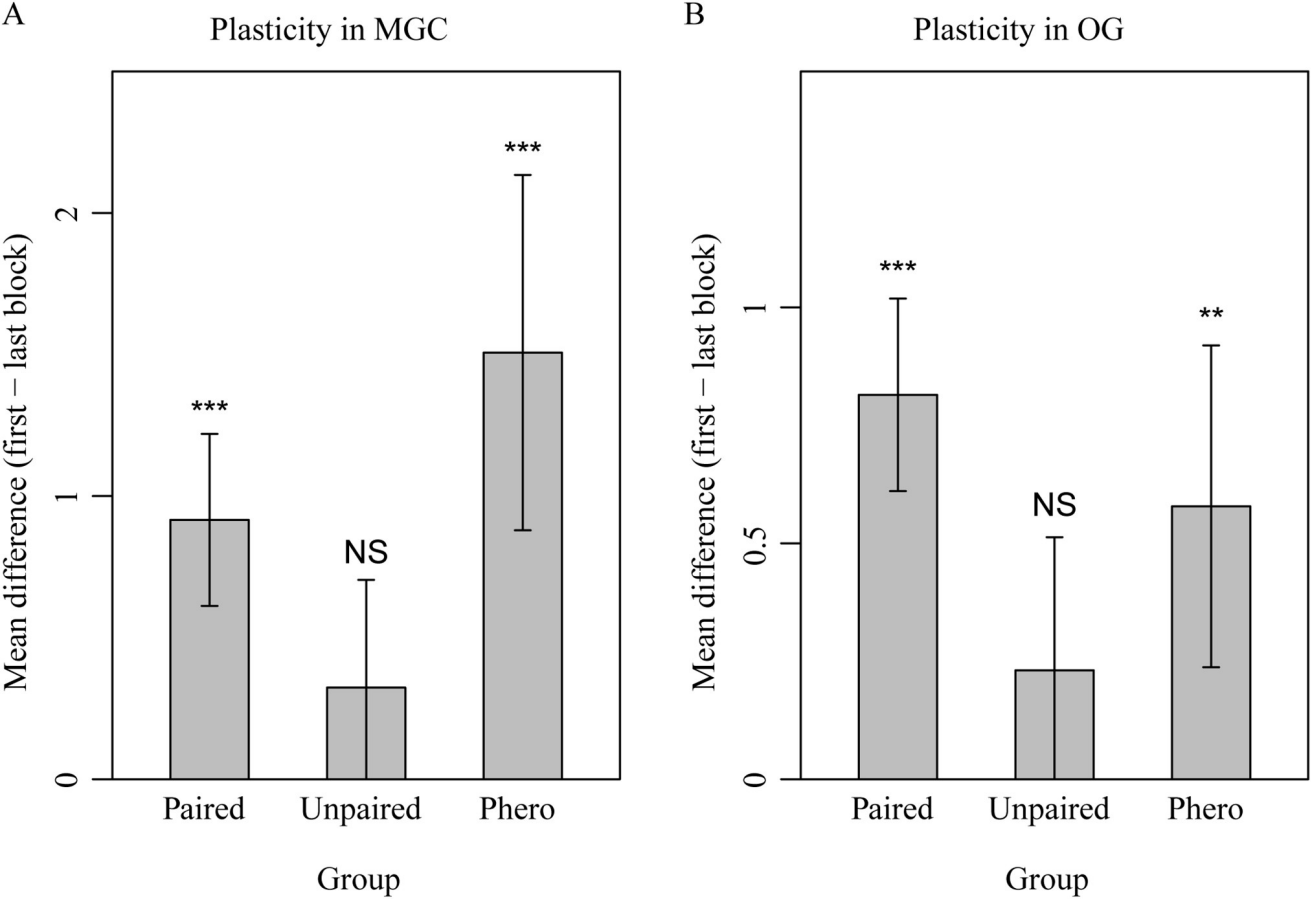
Mean ± 95CI of difference in response strengths between first and last repetition of the pheromone-paired plant blend, the unpaired plant blend, and the pheromone alone. Repetitive stimulation with paired pheromone-plant odor resulted in suppressed responses to both the pheromone and to the plant odor which was paired with it, but not to the unpaired plant odor. This suppression (positive value in the plot) was present in the MGC when averaged over glomeruli (A) and in the OG (B), indicating plastic changes in the AL network. Asterisks indicate changes from first to last repetition that are statistically larger than zero (paired t-tests: p < 0.05, ‘**’ p < 0.01, ‘***’ p < 0.001, ‘NS’ Not Significant).

Since, as previously mentioned it was not feasible to map each ordinary glomerulus across individuals, we pooled the responses from these units and analyzed the aggregated data. In the OG, as expected, pheromones elicited only weak responses compared to the plant odors (Fig 5C and 5D). A more detailed inquiry revealed that the pheromone typically elicited weak to moderate responses in a few glomeruli (typically 1 or 2 units per individual), whereas the plant odors each elicited moderate to strong responses in 4–8 glomeruli (data not shown). The pheromone-plant odor mixture also induced strong responses in the OG, albeit reduced compared to the respective plant blend on its own (Fig 5C and 5D). This reduction was apparent over the four repetitions and indicated that the pheromone had a suppressive effect on OG responses. Thus, activity in the MGC appears to inhibit the OG, and vice versa.We observed a decreased response strength in the OG and two of the MGC units, the cumulus and the dmp, during repetitive compound stimulation (pheromone + plant odor). Repetitive stimulation with plant odor alone, on the other hand, did not result in a significant reduction with the exception of sunflower in cumulus, where slight inhibitory responses were recorded in the later repetitions (Fig 5). Strikingly, after the repeated stimulation with pheromone-plant odor mix, responses to the pheromone and to the previously pheromone-paired plant odor on its own were reduced compared to before the pairing (6^th^ repetition vs 1^st^ repetition). This was apparent for both the MGC and the OG (Fig 5). Decreased responses were observed in the cumulus and the dmp, with the latter showing the most pronounced change (dmp, Figs 4 and 5). This glomerulus showed the strongest initial response to the pheromone as well. Stimulation with the unpaired plant odor did for the most part not result in the same response reduction, thus excluding that the observed effect in the plant-peromone mix responses was due to general effects like dye bleaching or adaptation (Fig 5). Pooled over both protocols (sunflower or ylang-ylang as pheromone-paired odor) and glomeruli, the sustained suppression as indicated by the difference in average response strength between first and last stimulus repetition was significant in the MGC (t-test_pheropaired_: t = 6.12, df = 35, p-value = 5.37e-07; t-test_phero_: t = 4.91, df = 29, p-value = 3.26e-05; t-test_unpaired_: t = 1.73, df = 35, p-value = 0.093) and the OG (t-test_pheropaired_: t = 7.91, df = 109, p-value = 2.23e-12; t-test_phero_: t = 3.37, df = 94, p-value = 0.0011; t-test_unpaired_: t = 1.62, df = 109, p-value = 0.1077) (Fig 6A and 6B, respectively).

### Divergence of odor response patterns as a consequence of sustained suppression

**Fig 7:**
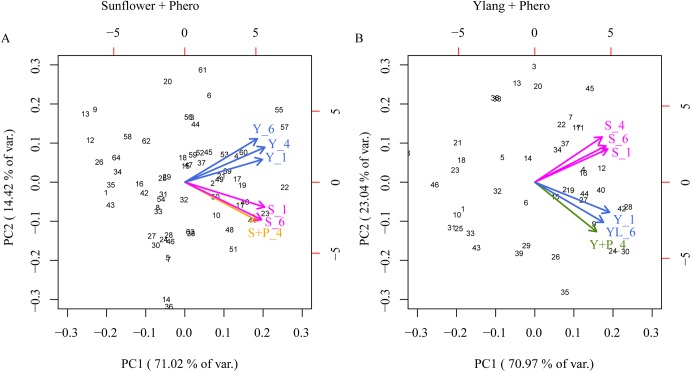
Principal component analysis of odor patterns in the OG of the AL reveals that the response patterns to sunflower and ylang-ylang, respectively, are more separated after the pheromone pairing than before. This occurred both when sunflower and ylang-ylang was paired with pheromones (A and B, respectively). The biplot contains scores of each ordinary glomerulus (black numbers) and loading of each odor pattern (colored vectors) on the first 2 principal components (explaining 84–94% of the variance).

Since suppression of weaker responses is a phenomenon known to enhance signal contrasts in networks containing lateral inhibition, like the AL, we further investigated the separation of odor response patterns with a principle component analysis of the PN data. The resulting loadings from the PCA revealed that the response patterns in the OG to the two plant odors diverged when one of the odors was paired with the pheromone, and confirmed that this divergence was still present at the end of the experiment where both plant odors were presented without pheromone (Fig 7).

This pattern separation was observable in both tested plant odor—pheromone combinations.

**Fig 8:**
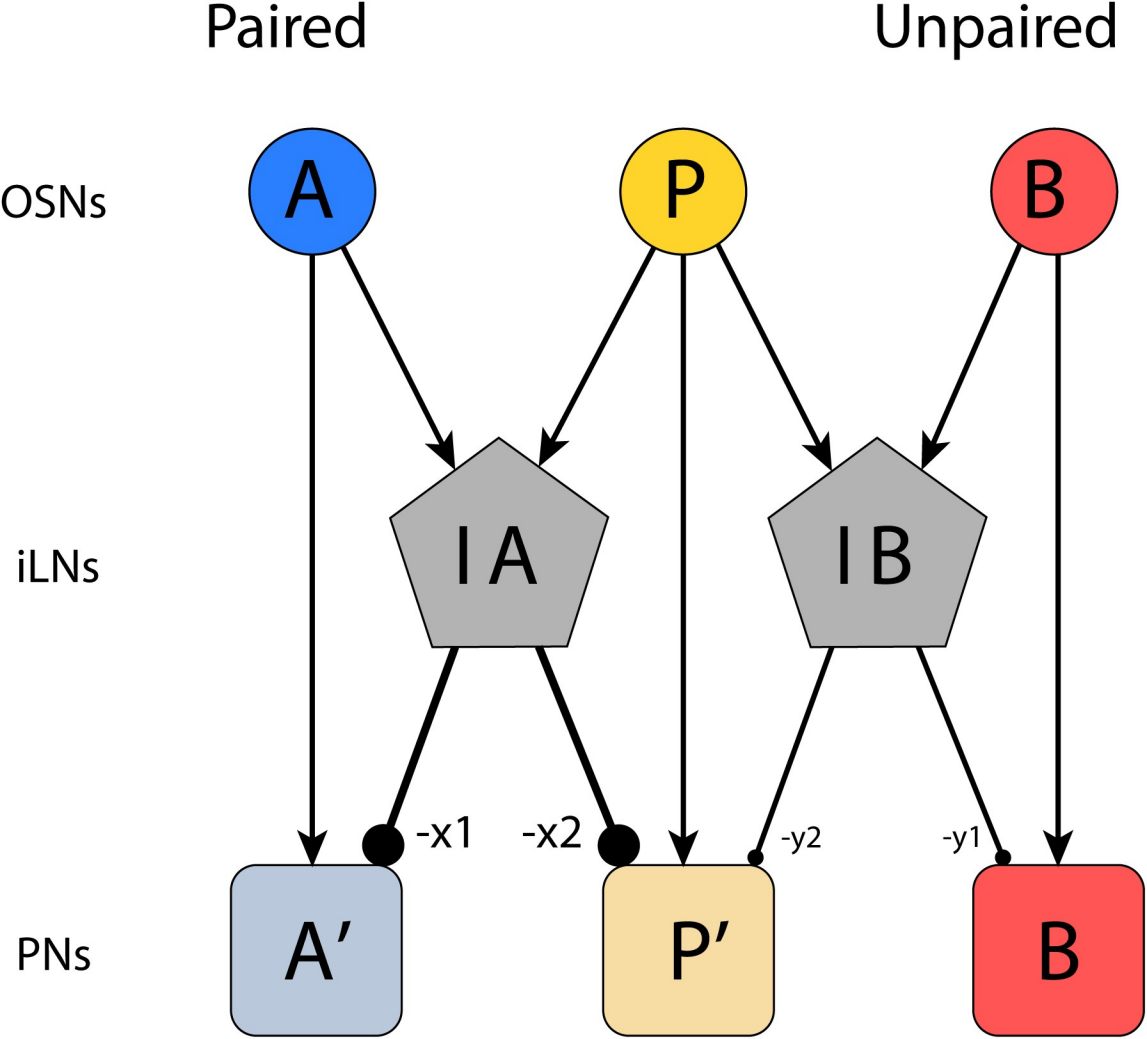
Model of putative AL network explaining how sustained suppression can be established by plant odor and pheromone interactions. In the situation where a plant odor (A) is paired with a pheromone (P) stimulus, corresponding OSNs (A + P) are activated simultaneously, which drives strong activation of inhibitory LNs (IA) such that the resulting excitation of PNs in the OG (A) and in the MGC (P) is suppressed compared to when the plant odor or the pheromone appears alone. The strong coincidental activation of the LNs (IA) causes a strengthening of the inhibitory synapses (x1 and x2), such that responses to subsequent stimuli with the plant odor (A) or the pheromone (P) are suppressed. In the situation where a plant odor (B) is presented alone without the pheromone, the resulting activation of connected LNs (IB) is weaker. Thus, strengthening of the inhibitory synapses onto the PNs does not occur (y1 and y2), leaving the PN responses unaltered.

We propose that the observed plasticity following repeated presentation of paired pheromone and plant odor are caused by up-regulation of inhibitory synapses in inhibitory local interneurons (iLNs) which operate between the MGC and the OG (Fig 8).

Since the suppression in the OG was only observed for the responses to the pheromone-paired plant odor, we hypothesize that the coincidental input from both plant odor and pheromone OSNs are necessary to trigger the synaptic modification. Thus, plant odors and pheromone signals without temporal overlap do not elicit this interaction-dependent plasticity.

In summary, we observed a suppression effect both in the MGC and the OG when applying a mixture of plant odor and pheromone to the insect antenna, and we found that this suppression increases after repeated stimulation, indicating synaptic plasticity. Moreover, the suppression was maintained in both regions after repeated stimuli of the pheromone-plant odor mixture, when applying each of the paired odorants alone (Fig 6). A general consequence of the suppression effect in the OG was that the response patterns to the two relevant plant odor blends became more separated after systematically pairing each blend with the pheromone (Fig 7).

## Discussion

The pathways for pheromones and plant odors are segregated in two parallel olfactory sub-systems in insects. In the moth, this segregation is particularly prominent in the male AL in shape of two distinct structures: a male-specific MGC and an assembly of ordinary glomeruli–both formed by neural branches of input and output neurons. In the study presented here we succeeded in performing retrograde staining of the uni-glomerular PNs confined to the mALT by applying a calcium sensitive dye in the calyx of the mushroom bodies. We could then map odor-evoked responses in this distinct population of AL output neurons, including neurons of both sub-systems, via calcium measurements from the AL glomeruli. As expected, we found that antennal stimulation with the species-specific pheromone elicited excitatory responses mainly in the three MGC glomeruli whereas application of the two plant odor blends (sunflower headspace and ylang-ylang) induced responses primarily in the OG. In addition, we found that stimulation with one of the plant odor blends combined with the pheromone led to both a suppressed response in the OG as compared to that induced by the plant odor alone and a suppressed response in the MGC as compared to that induced by the pheromone alone. Importantly, we found that these suppressions were increased by repeated stimulation, and led to a reduced response even when the previously paired odor stimuli were later presented alone. The fact that the response to the other, unpaired plant odor did not decrease, in spite of being equally repeatedly presented, suggests that a persistent interaction between the neural networks linked to the MGC and the OG takes place during concurrent stimulation with the pheromone and plant odor.

The finding that antennal stimulation with the binary pheromone induced responses in the three glomeruli constituting the MGC of *H*. *armigera* is in general agreement with previous investigations. In a recent calcium imaging study on this particular species including the bath application technique it was reported that stimulation with the major pheromone component, Z11-16:AL, elicited responses in the cumulus whereas the second component, Z9-16:AL, activated the dorsomedial posterior MGC glomerulus (dmp) [23]. Two former calcium imaging studies on the related heliothine species, *Heliothis virescens*, have reported corresponding findings including responses in distinct MGC units during antennal stimulation with each of the single pheromone components [24, 25]. These previous calcium imaging studies applied the bath application technique, which is assumed to map the input by primarily reflecting OSN activity. In the current study, however, we could determine the output signals, based on measurement of neural activity in selectively labeled PNs. Interestingly, we found responses not only in all three MGC units–the cumulus, the dmp, and the dorsomedial anterior glomerulus (dma) during stimulation with the binary pheromone blend–but also a stronger response in the dmp to the pheromone than in the two remaining MGC glomeruli. Whether the dmp, being known to receive input about the secondary pheromone component [23], might be a specific site for synergistic PNs carrying integrated pheromone information to the higher brain centers is an open question. Two previous publications containing data from tracing of physiologically identified PNs in *H*. *virescens* have indeed reported about uni-glomerular PNs confined to the MGC-unit receiving input about the second pheromone component that responded synergistically when stimulated with the binary pheromone blend [26, 27].

Stimulation with each of the two natural plant odor blends, sunflower headspace and ylang-ylang, evoked responses in several OG demonstrating activation patterns having a substantial degree of overlap. The fact that each plant odor stimulus tested here contains numerous compounds, several of which are known to serve as ligands for distinct types of OSNs, is most likely one reason for these complex responses [18]. Another issue is the fact that also pure chemical compounds are reported to activate AL output neurons originating not only from one but from several glomeruli, as shown in other species [28, 29].

We found that the response in the sexually isomorphic glomeruli was gradually suppressed when a combination of one plant odor blend and the pheromone was given repeatedly, both for the sunflower headspace and the ylang-ylang stimulus. Conversely, we found that responses to the plant odor alone remained stable over the same time period and number of stimulations. Together, these two observations suggest that the altered response was established due to the simultaneous activation of both sub-systems, indicating Hebbian-like plasticity. The fact that the change consists in a reduced response seems indeed appropriate. Probably, it is beneficial for the male moth to be able not only to detect a plant odor occurring together with the pheromone there and then, but also to easily recognize it later on, possibly when being presented alone. An appropriate way of discriminating between many different plant odor blends initially being widely represented in the AL, and thus having overlapping activation maps, is to fine tune the response patterns of the important ones and thereby making them more easy to distinguish from others. The general interaction between the male-specific and the plant odor system in moths is clearly demonstrated via numerous former investigations, including behavioral, electrophysiological, and calcium imaging studies. Previous behavioral tests on heliothine moths have, for example, reported about green leaf volatiles, such as *cis*-3-hexenyl acetate, enhancing the male’s sexual attraction when added to the pheromone of the conspecific female [12, 30]. Furthermore, electrophysiological studies on the sphinx moth, *Manduca sexta*, have shown that activation of the MGC dampens PN responses in the OG [31, 32]. Most previous physiological investigations have explored how plant odors influence pheromone responses. Data from these studies vary considerably, some reporting about enhanced responses in OSNs [6, 11], or in PNs [10]. Other studies report about reduced responses at receptor level [8], OSNs [7], or PNs [9, 33]. Notably, a recent publication on *H*. *virescens* showed that pheromone-plant odor interactions in *H*. *virescens* might be an effect of stimulation with supra-natural plant odor concentrations [20]. Therefore, in the study presented here we applied lower concentrations than those previously used.

There is a general agreement that the population of AL LNs plays a key role in establishing interactions between the two olfactory sub-systems of the male moth. Previous morphological studies, performed on *B*. *mori*, have reported about a major group of LNs innervating both the MGC and the OG [34]. The fact that this group includes both multi- and oligo-glomerular LNs suggests that these neurons have various functions. In general, multi-glomerular LNs are suggested to serve numerous roles including global gain control and lateral inhibition/excitation [35, 36], whereas oligo-glomerular LNs may function as distinct networks maintaining signal processing about behaviorally important odor blends [32, 37, 38]. Physiological data from LNs are relatively limited but in *M*. *sexta* it was found that a majority of those neurons being activated by the pheromone showed inhibitory responses [32]. As in other insects, most LNs in the moth AL are GABAergic [39] and in *H*. *virescens*, the number is estimated to be approximately 330 [40]. The neural plasticity observed in the present study might be caused by strengthening of inhibitory synapses between PNs and global inhibitory LNs (Fig 8). The fact that the reduction effect was observed only for paired stimuli indicates the necessity for the coinciding input from the plant odor and the pheromone OSNs. Thus, plant odor and pheromone signals without temporal overlap were not subjected to this interaction-dependent plasticity.

Synaptic plasticity in insects has been observed, so far, in classical conditioning experiments, when an unconditioned stimulus (US, e.g. a sugar reward) is paired with a conditioned stimulus (CS, e.g. an odor). Previously, a form of non-associative plasticity in the vertebrate olfactory bulb has been demonstrated, where the glomerular activation map became more focused after several seconds of stimulation [41]. Here however, we report a form of Hebbian-like plasticity that involves the temporal pairing of two odors. Most likely, this novel type of plasticity is more related to olfactory coding, i.e. the capacity of the system to identify and process particular odors, rather than valence attribution, i.e. learning that an odor predicts something. In bees, it has been shown that classical conditioning creates, in parallel, an associative memory trace and a sensory memory trace, the latter being dependent on gene methylation [42, 43]. Here we show, to our knowledge for the first time in insects, that by pairing two odors in time, it is possible to induce a sensory memory trace without the need for appetitive or aversive conditioning.

## Supporting information

S1 Fig **Quantile-quantile plots of residuals for response strength in MGC (A) and OG (B) for paired pheromone-plant odor (left), unpaired plant odor (middle), and pheromone (right) responses.** The residuals fall within the confidence bounds expected for normally distributed data, justifying the use of parametric statistics.
(TIF)

S1 Data Collective response data tables and Imaging raw data files. Calcium response data from antenna lobe projection neurons in male moth (Helicoverpa armigera) stained with Fura2 (ratiometric), stimulated with plant odors (Sunflower or Ylang ylang) in presence with pheromones. Raw data Imaging files available at: https://figshare.com/articles/Imaging_rawdata_Trondheim_zip/4810420.
(ZIP)
