# Supplementary figures and images for: Coincidence of pheromone and plant odor leads to sensory plasticity in the heliothine olfactory system
Source: PLoS One. 2017 May 3;12(5):e0175513. doi: 10.1371/journal.pone.0175513 (PMC5414983; doi:10.1371/journal.pone.0175513)

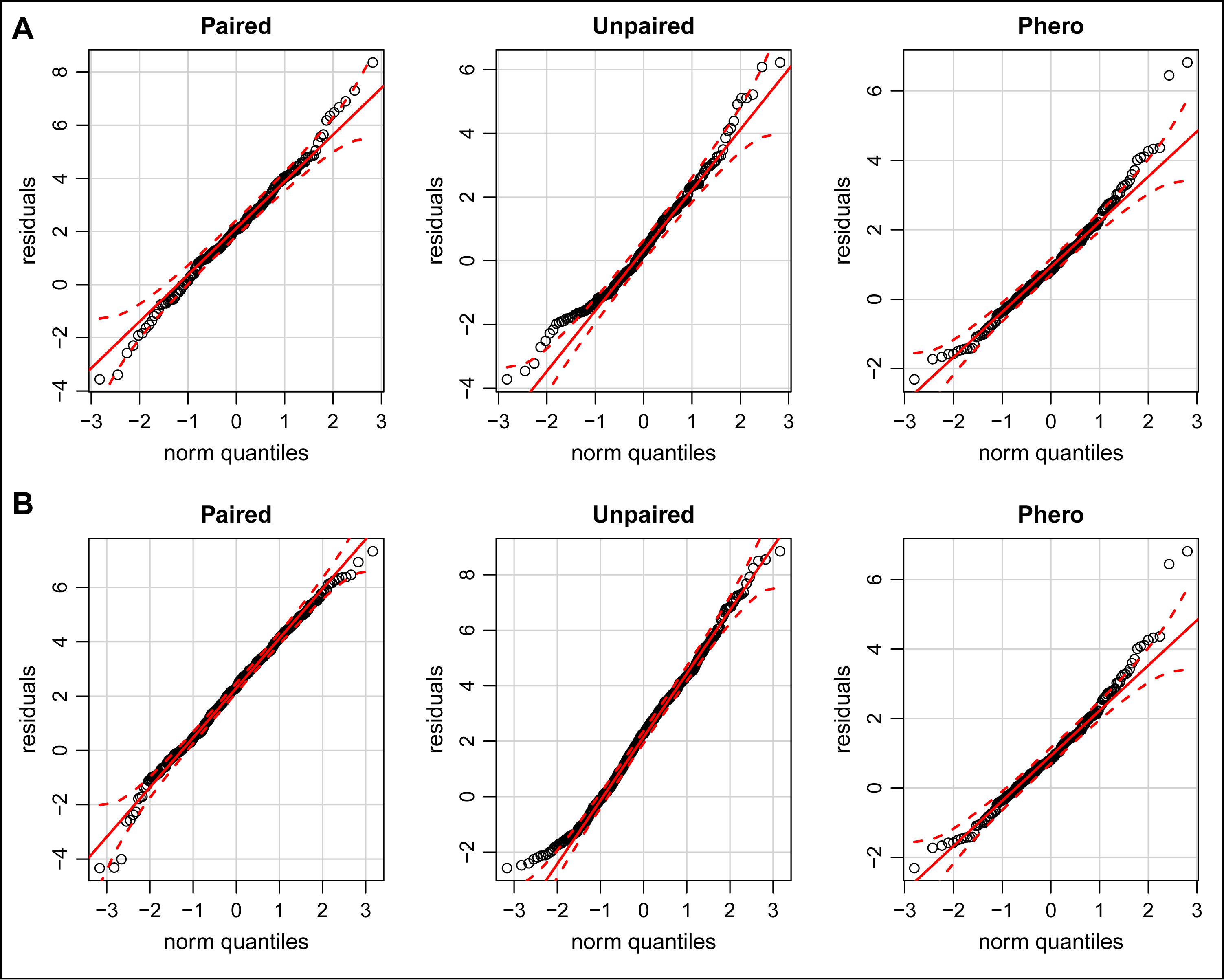

Supplement: S1 Fig — Quantile-quantile plots of residuals for response strength in MGC (A) and OG (B) for paired pheromone-plant odor (left), unpaired plant odor (middle), and pheromone (right) responses. The residuals fall within the confidence bounds expected for normally distributed data, justifying the use of parametric statistics. (TIF) [file pone.0175513.s001.tif]
